# Antibiotic resistance of Gram-negative benthic bacteria isolated from the sediments of Kardzhali Dam (Bulgaria)

**DOI:** 10.1080/13102818.2014.998160

**Published:** 2015-01-20

**Authors:** Ivan Iliev, Mariana Marhova, Velizar Gochev, Marinela Tsankova, Sonya Trifonova

**Affiliations:** ^a^ Department of Biochemistry and Microbiology, Faculty of Biology, University of Plovdiv “Paisii Hilendarski”, Plovdiv, Bulgaria

**Keywords:** antibiotic resistance, Gram-negative microflora, fish farm, sediments

## Abstract

The aim of the present study was to carry out a preliminary assessment for the occurrence of bacterial strains resistant to frequently used antibiotics in the sediments beneath the sturgeon cage farm in Kardzhali Dam (Bulgaria). Samples were taken from the top 2 cm of sediments under a fish farm and from a control station in the aquatory of the reservoir in the period July–October 2011. Surveillance of bacterial susceptibility to 16 antimicrobial agents was performed for 160 Gram-negative strains (*Pseudomonas mandelii* – 100 strains; *Hafnia alvei* – 30 strains; and *Raoultella ornithinolytica* – 30 strains). No significant differences in the resistance to the tested antibiotics were observed between the strains isolated from the two stations (analysis of variance, *P* > 0.05). Widespread resistance to penicillins and certain cephalosporin antibiotics was observed in both stations. None of the studied strains showed resistance to the aminoglycoside antibiotics gentamicin and amikacin, or to ciprofloxacin. Minimal Inhibitory Concentrations (MIC) were determined for five of the tested antimicrobial agents by the microdilution antibiotic sensitivity assay. The data indicate that amikacin, tetracycline and ciprofloxacin effectively suppress the growth of the tested micro-organisms. The isolates from genus *Pseudomonas* showed the highest MIC and were characterized by the highest percentage of antibiotic resistance.

## Introduction

Antimicrobial agents have a wide range of applications for control and prevention of infectious diseases in humans and animals.[1] The increasing resistance of micro-organisms to used antibiotics is globally acknowledged as a serious ecological problem.[2–5] Antimicrobial resistance is a direct consequence of the misuse of antibiotics in veterinary medicine and aquaculture, causing selective pressure on bacterial species.

Net cage fish farms have a direct impact on the aquatic environment. Usually, medicated feed is used for antibiotic treatment of fish. Contamination of the environment with drugs occurs due to release of uneaten feed and faeces in the water column.[1] One of the challenges for the development of cage aquaculture in open waters is disease caused by pathogenic bacteria such as *Aeromonas* spp., *Vibrio* spp., *Pseudomonas* spp. and *Flavobacterium* spp.[1,6] This can result in propagation of resistant strains in the environment.[4,7] Such high frequency of occurrence of bacterial resistance is well documented in clinical isolates and in wild animal populations and natural water samples. Allochthonous and antibiotic-resistant pathogenic bacteria introduced in the environment can transfer their resistance to autochthonous aquatic bacteria, which can in turn transfer it to susceptible autochthonous pathogenic bacteria.[4,8–11]

Although fish farms have existed since the 1960s in Europe, this problem is poorly documented.[1,12,13] Today, due to the development of good practices for sustainable management of land and freshwater use, it seems necessary to enhance that knowledge.

The aim of this study was to carry out a preliminary assessment for the occurrence of bacterial strains resistant to frequently used antibiotics in the sediments beneath the sturgeon cage farm in Kardzhali Dam (Bulgaria).

## Materials and methods

### Study area

**Figure 1.**
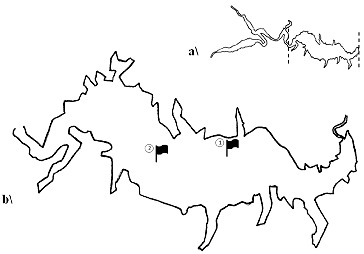
General scheme of Kardzhali Dam (a) and locations of sampling stations (b) in the studied area of the reservoir.

The study was carried out in the aquatory of Kardzhali Dam in the period July–October 2011. Two sampling stations were selected on the basis of a preliminary survey on the spatial impact of cage farms. Station 1(N 41°38′27″; E 25°18′43″) is located near a cage fish farm which has been used for commercial rearing of carp and sturgeon species for approximately 30 years. Station 2 (N 41°38′44″; E 25°17′37″) is situated in the upper part of the reservoir, which is free of cages, and was used as a control station. The exact location was determined by a Garmin 76CS x GPS receiver (Figure 1).

### Sediment sampling

Sediment samples were collected from the bottom with an Ekman–Birge bottom sampler. The upper two centimetres from the sediment core were transferred into sterile plastic tubes and transported on ice to the laboratory within six hours. Samples were taken three times for the period of the study in order to compare the results from the control and cage farm station.

### Tested strains

After identification, a total of 50 strains of *Pseudomonas mandelii*, 30 strains of *Raoultella ornithinolytica* and 30 strains of *Hafnia alvei* isolated from the sediments of station 1 and 50 strains of *Pseudomonas mandelii* isolated from station 2 were tested for antibiotic sensitivity.

### Antibiotic sensitivity assay

The antibiotic susceptibility testing of all isolates was carried out using the disc-diffusion method of Bauer–Kerby.[14] Sixteen antimicrobial agents were selected as representatives of seven different classes of antibiotics used in aquaculture: ampicillin (AMP, 10 µg), ampicillin/sulbactam (SAM, 20 µg), amoxicillin (AML, 10 µg), amoxicillin/clavulanic acid (AMC, 30/15 µg), cefuroxime (CFX, 30 µg), cefixime (CFM, 5 µg), cefoperazone/ sulbactam (CFS, 75/35 µg), cefalothine (KF, 30 µg), amikacin (АК, 30 µg), gentamicine (GN, 10 µg), tetracycline (TE, 30 µg), erythromycin (Е, 15 µg), ciprofloxacin (CIP, 5 µg), nalidixic acid (NAL, 30 µg), sulfamethoxazole/trimethoprim (SXT, 23.75/1.25 µg) and chloramphenicol (C, 30 µg). All were products of Oxoid (Hampshire, England). Reference strains *Escherichia coli* ATCC 25922 and *Pseudomonas aeruginosa* ATCC 27853 were used as internal standards.

### Determination of minimal inhibitory concentration

The minimal inhibitory concentration (MIC) of five antimicrobial agents (tetracycline, amikacin, ciprofloxacin, nalidixic acid and sulfamethoxazole) was determined by the microdilution antibiotic sensitivity assay, according to the Clinical and Laboratory Standards Institute.[14] After 18–24 h of incubation on Mueller–Hinton agar at 37 °C, a single colony from each strain was transferred into Mueller–Hinton Broth (HiMedia) and adjusted to optical density (OD) 0.5 McFarland units. Antibiotic stock solutions with a final concentration of 1280 µg∙mL^−1^ (products of HiMedia, India) were prepared in Mueller–Hinton Broth. Assays were conducted in 96-well microtiter plates at 10 different concentrations for each antimicrobial agent prepared by series of two-fold dilutions (64–0.125 µg∙mL^−1^). Higher concentrations were used for sulfamethoxazole (640 –1.25 µg∙mL^−1^). Prior to inoculation, the standardized bacterial suspensions were diluted 1:100 and 100 µL were added to each well containing 100 µL of the tested antimicrobial agent. This resulted in a final inoculum of 5 × 10^5^ cfu∙mL^−1^.[14] MIC values were determined after 24 h incubation at 37 °C. The turbidity of each well was measured by an ELx 800 spectrophotometer (Bio-Tek). RidaWin V 1.31 software (R-Biopharm AG) was used to analyse the results. MIC was defined as the lowest concentration of the antimicrobial agent that inhibits the growth of the tested isolate more than 80% compared to that in the positive control well.

### Statistical analysis

Statistical analysis was performed with Statistica V 10 (StatSoft). Analysis of variance (ANOVA) was used for sample comparison in order to examine the null hypothesis that antibiotic resistance in bacteria isolated from sediments under the fish farm was greater than in isolates from the control sediments.

## Results and discussion

### Antibiotic sensitivity of Gram-negative microflora

A total of 160 Gram-negative strains isolated from sediments at stations 1 and 2 were tested for sensitivity to 16 antibiotics. Gram-negative species were used as a more reliable subject for testing antibiotic resistance due to their marked resistance to a broad spectrum of antibiotics, as opposed to Gram-positive micro-organisms.[15,16] The gram-negative microflora in the sediments beneath the two stations was formed mainly by *Pseudomonas mandelii.* This was the only species isolated from station 2. At station 1, the species composition included representatives of the coliform group, *Raoultella ornithinolytica* and *Hafnia alvei*.

Widespread resistance for penicillins, some cephalosporin antibiotics and erythromycin was observed. All isolated strains were found to be resistant to AMP, AMX and E; 79% were resistant to SAM, 81% to AMC and KF, 75% to CFX; 62% to CFM and C; 59% to CFS; 12.5% to TE and NAL, and 5% to cotrimoxazole. There was no established resistance of the studied strains to the aminoglycoside antibiotics GN and AK, as well as to CIP. The reference strain *Escherichia coli* ATCC 25922 was sensitive to all the antimicrobial agents tested, with the exception of erythromycin. *Pseudomonas aeruginosa* ATCC 27853 showed resistance to AP, SAM, AML, AMC, CXM, CFM, KF, E, NAL, C and SXT. It was susceptible to CES, AK, G and CIP. The results for the reference strains are in accordance with the acceptable limits for quality control strains used to monitor the accuracy of disk diffusion testing of nonfastidious micro-organisms.[14]

Resistance to tetracycline and tetracyclines is a common phenomenon.[17–19] However, only 21 *Pseudomonas mandelii* strains that were isolated from the sediments from Kardzhali Dam were resistant to tetracycline. Resistance to tetracycline was not observed in *H. alvei* and *R. ornithinolytica*. Tetracyclines are characterized by a broad spectrum of action and resistance to them is often plasmid-determined. The presence of strains resistant to tetracycline poses risk of transmission of this resistance to pathogenic species.[20]

Resistance to quinolones is rare within representatives of the natural microflora and does not exceed 25%,[12] although Chelossi et al. [20] have found that 70% of their isolates were resistant to nalidixic acid. These levels are much higher compared to the results obtained for Kardzhali Dam. We determined the resistant strains to be 12.5% of all tested isolates. For *P. mandelii*, the level of resistance was higher and reached 21%. None of the tested strains was found to be resistant to ciprofloxacin. It must be taken into account, however, that neither of these substances is approved for use in aquaculture.

#### Pseudomonas mandelii

**Figure 2.**
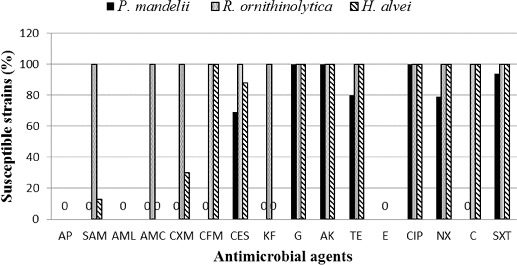
Sensitivity of the 160 Gram-negative strains isolated from the sediments of Kardzhali Dam (*P. mandelii* – 100 strains; *H. alvei* – 30 strains; and *R. ornithinolytica* – 30 strains).

Note: AMP – ampicillin; SAM – ampicillin/sulbactam; AMX – amoxicillin; AMC – amoxicillin/clavulanic acid; CXM – cefuroxime; CFM– cefixime; CES – cefoperazone/sulbactam; KF – cephalothin; G – gentamicin; AK – amikacin; TE – tetracycline; E – erythromycin; CIP – ciprofloxacin, NAL – nalidixic acid; C – chloramphenicol; SXT – sulfamethoxazole/trimethoprim.

All of the 100 tested strains showed resistance to ampicillin and amoxicillin, irrespective of the addition of *β*-lactamase inhibitors such as sulbactam and clavulanic acid, as well as to cephalosporins CFM, CFX and KF (Figure 2). A total of 31 (31%) strains were resistant to cefoperazone with added sulbactam: 19 of those isolated from station 1, and 12 from the sediments at station 2. Eight of the isolates from station 1 and 12 strains from station 2 demonstrated resistance to TE (20%), while resistance to NAL was observed in 21 strains (21%), eight of which were isolated from station 1 and 13, from station 2. Resistance to cotrimoxazole was shown in four strains (4%) from the sediments at station 1. There was no resistance to GN, AK and CIP in the tested *P. mandelii* strains. No significant differences were found in the resistance of the strains isolated from stations 1 and 2 to the tested antibiotics (ANOVA, *P* > 0.05).

Of the identified bacterial groups, the representatives of genus *Pseudomonas* are notorious for their resistance to antibiotics, as these bacteria maintain antibiotic resistance plasmids.[21] Since these plasmids are transmissible, the increased resistance to certain antibiotics poses a threat of transferring such resistance to sensitive bacteria, thus making them acquire resistance to antibiotics.[16,22]

#### Raoultella ornithinolytica

Among the 30 strains tested, 100% resistance to AMP and AMX was established. The addition of *β*-lactamase inhibitors decreased the resistance to 0%. All strains were sensitive to first-, second- and third-generation cephalosporins, to aminoglycoside antibiotics (AK and GN), to quinolones (CIP and NAL) and to sulfonamides (cotrimoxazole). Only the *R. ornithinolytica* isolates were sensitive to chloramphenicol, which is one of the main agents used in aquaculture. In our study, 62% of the total strains tested showed resistance to chloramphenicol. Resistance of Gram-negative micro-organisms to chloramphenicol was reported in Chili [23] and France [24]. At present, due to its proven cytotoxicity, this substance is banned in the European Union.[25]

#### Hafnia alvei

Isolates of *H. alvei* exhibited higher resistance to the tested antibiotics compared to *R. ornithinolytica*. All 30 strains (100%) were resistant to AMP, AMX, AMC, KF and E. Full susceptibility (100%) was observed to CFM, GN, AK, TE, CIP, NAL and cotrimoxazole. Six strains (20%) were resistant to SAM and nine strains (30%), to CFX. Four strains (13%) showed resistance to KF.

The level of resistance of coliforms to the tested antimicrobial agents was low, with no reported evidence for the emergence of such resistance in the strains in our study. They exhibited higher sensitivity to ampicillin and ciprofloxacin in comparison to results obtained from research of waste water.[3,26] What is essential for the emergence of resistant forms of microorganisms is the presence of favourable conditions for them to divide.[27] In the bottom layers of Kardzhali Dam the temperatures are low and create unfavourable conditions for reproduction of *Enterobacteriaceae* species in the sediments.

The results from the tests for antimicrobial activity did not show significant differences in respect to the number of resistant strains isolated from the two stations (ANOVA, *P* > 0.05). Unlike our results, Gordon et al. [1], who studied the effect of net-cage farms on the counts of representatives of genus *Aeromonas* and genus *Pseudomonas* in the sediment under the farms, observed a significant increase in the number of *Aeromonas* spp. resistant to OA (oxolinic acid) and OTC (oxytetracycline) during the period of active feeding, without this affecting the adjacent areas.

Our study showed high levels of antibiotic resistance of the isolates from sediments from the two stations. The levels of resistance do not differ significantly from those reported in similar researches in countries where the use of antibiotics in aquaculture is proven. The strains we tested exhibited resistance to AMP, AMX, first- and second-generation cephalosporins and erythromycin. The resistance of Gram-negative micro-organisms to erythromycin in the present study is consistent with reports by other authors.[6,17,18,20]

**Figure 3.**
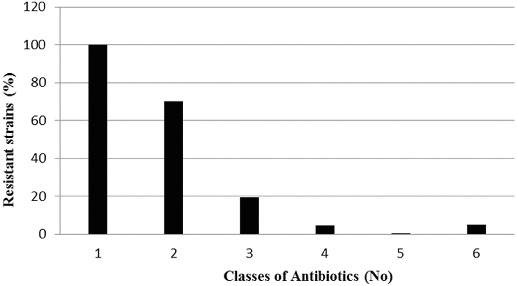
Multiple antibiotic resistance of Gram-negative strains isolated from the sediments of Kardzhali Dam in August 2011.

The analysis of the results shows that eight *P. mandelii* strains (5%) were characterized with resistance to six of the eight tested classes of antibiotics. Resistance to four of the tested classes was established in 7.5% of the cases (12 *P. mandelii* strains), and 31 *P. mandelii* isolates were resistant to three classes. There was resistance to two of the classes in 70% of the strains, including all *P. mandelii* isolates and 12 *H. alvei* isolates. In all (100%) of the tested strains, there was resistance to at least one class of antibiotics (Figure 3).

The study showed resistance to more than one class of antibiotics in 68% of the tested isolates. Multiple resistance is a characteristic trait reported in a number of studies on pathogenic Gram-negative micro-organisms in aquaculture.[28] The natural microflora in water basins rarely presents a threat of causing infectious diseases in the inhabitants. The development of resistance to a given group of antibiotics in representatives of the natural microflora poses a risk of creating a pool of resistant genes which can be transferred in transposable elements.[29] This entails a significant risk of emergence of pathogenic strains of micro-organisms with broad-spectrum antibiotic resistance.[4]

### Minimum inhibitory concentration

**Figure 4.**
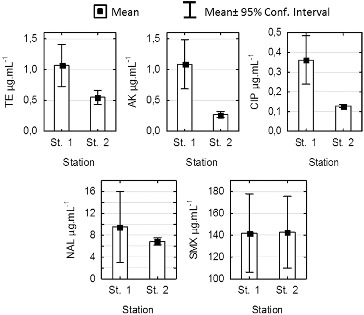
Comparison of minimum inhibitory concentration (MAC) for *Pseudomonas mandelii* strains isolated from sediments at stations 1 and 2 in Kardzhali Dam in August 2011.

Note: TE – tetracycline; AK – amikacin; CIP – ciprofloxacin, NAL – nalidixic acid; SMX – sulfamathoxazole.

The minimum inhibitory concentration (MIC) values of tetracycline, amikacin, ciprofloxacin, nalidixic acid and sulfamethoxazole for 60 tested strains of *P. mandelii* isolated from the sediment in stations 1 and 2 are shown in Figure 4. The data indicate that AK, TE and CIP effectively suppress the growth of the tested micro-organisms, and the reported MIC values correspond to the standards of sensitivity for *Pseudomonas aeruginosa*.[14]

The MIC values for nalidixic acid indicated average levels of sensitivity and in 6 of the studied 30 strains from station 1, they exceeded the set standards for sensitivity of 16 μg·mL^−1^.[6] For station 2, the average value was 6.85 μg·mL^−1^ and did not exceed 8 μg·mL^−1^. The isolates that showed the highest MIC were those from genus *Pseudomonas*. They were also characterized by the highest percentage of antibiotic resistance.

The statistical analysis showed significant differences between the MIC values in stations 1 and 2 for tetracycline, amikacin and ciprofloxacin (ANOVA, *P* < 0.005). There were no differences in the sensitivity between the two stations for NAL and sulfamethoxazole. The mean MIC values for SMX of about 140 μg·mL^−1^ indicate that the investigated strains are not sensitive to SMX.

**Figure 5.**
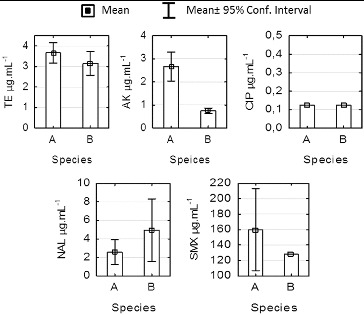
Minimum inhibitory concentration for strains *Hafnia alvei* (a) and *Raoultella ornirhinolytica* (b) isolated form station 1sediments at Kardzhali Dam in August 2011.

Note: TE – tetracycline; AK – amikacin; CIP – ciprofloxacin, NAL – nalidixic acid; SMX – sulfamathoxazole.

The results from the MIC test for the *Enterobacteriaceae* representatives isolated at station 1 are shown in Figure 5. The mean MIC values for TE were 3.6 and 3.2 μg·mL^−1^, respectively, for *H. alvei* and *R. ornithinolytica*. These results were consistent with the standards for MIC in the *Enterobacteriaceae* family.[14] *H. alvei* isolates demonstrated high sensitivity to TE, AK and CIP. All strains showed higher MIC values for AK as compared to *R. ornithinolytica* (ANOVA, *P* < 0.05) with low levels of intragroup variation, which is evidence for differences in the levels of sensitivity within the family. The average values for AK of 0.6 μg·mL^−1^ for *R. ornithinolytica* are comparable to the 0.5 μg·mL^−1^ reported by Szabо [30] for *Klebsiella pnumoniae*. No differences in the sensitivity to TE, CIP, NAL and SMX (ANOVA, *P* > 0.05) were observed in our study.

For SMX, the mean MIC values that we determined were 160 and 128 μg·mL^−1^ for *H. alvei* and *R. ornithinolytica* in station 1, respectively. These values are significantly lower than the ones reported by Wenz et al. [31]: 512 μg·mL^−1^ of SMX against *E. coli* and *Klebsiella* spp. The high values for SMX in the absence of cotrimoxazole-resistant strains of the two studied species indicate poor efficiency of SMX in the absence of a DHFR (dihydrofolate reductase) inhibitor, such as trimethoprim. The higher sensitivity to CIP than to NAL is in agreement with the results of Barry et al. [32], who demonstrated that CIP was four to eight times more active against Gram-negative representatives of family *Enterobacteriaceae* and *Pseudomonas* spp. compared to oxolonic and nalidixic acid with MIC values ≤ 2 μg·mL^−1^.

The lowest MIC values for all benthic isolates in our study were those for the antibiotics amikacin and gentamicin. Similar results have been obtained by Pontes et al. [4] for genus *Pseudomonas*.

The obtained results showed widespread resistance, which confirms that water micro-organisms are highly resistant to antibiotics.[20,28,33] It is indicative for the absence of selective pressure in the aquatory of the reservoir. Such a survey could be used as complement to the frequently used chemical monitoring.[1] This coupled approach would increase the reliability of the assessment of the ecological impact on the water body caused by the implementation of antibiotics in aquaculture. The extent of resistance found and the level of multiple resistance demonstrate the need of further studies on the effect of human activities in the area on the benthic microbial communities.

## Conclusions

Our analysis of the species composition of Gram-negative microflora in the sediments under the net cage farm and at the control station showed low diversity, with the main representative being *Pseudomonas mandelii*. Although the antibiotic susceptibility assay did not show any significant differences in the level of resistance between the isolates from the two locations, the results from the MIC test indicated higher antibiotic resistance of the strains isolated from beneath the cage farm. The high number of resistant strains isolated from fish-farm and control sediments suggests that antibiotic resistance is common in Gram-negative bacteria in the sediments of the studied area of Kardzhali Dam.

## Disclosure statement

No potential conflict of interest was reported by the authors.
